# Impact of a Mentored Peer-Review Program on the Sponsoring Journal: Program Evaluation at Five Years

**DOI:** 10.5811/westjem.64401

**Published:** 2026-10-21

**Authors:** Jeffrey N. Love, David P. Way, Wendy C. Coates, Anne M. Messman, Chris Merritt, Douglas S. Ander, Jonathan S. Ilgen

**Affiliations:** * Georgetown School of Medicine, Department of Emergency Medicine, Washington, District of Columbia; † Ohio State University College of Medicine, Department of Emergency Medicine, Columbus, Ohio; ‡ David Geffen School of Medicine UCLA, Department of Emergency Medicine, Los Angeles, California; § Wayne State University School of Medicine, Department of Emergency Medicine, Detroit, Michigan; ¶ University of Washington, Department of Emergency Medicine, Seattle, Washington; || Boston University Chobanian & Avedisian School of Medicine, Department of Emergency Medicine, Boston, Massachusetts; # Emory University School of Medicine, Atlanta, Georgia

## Abstract

**Introduction:**

The number of peer-reviewed biomedical journals has more than tripled over the past 20–25 years increasing the demand for quality peer reviewers. This demand and the lack of available formal training in peer review have resulted in a shortage of qualified peer reviewers. In response to this shortage, the *Western Journal of Emergency Medicine* Special Issue in Education (Special Issue) developed a formal mentored peer-review (MPR) program that provides a learning experience in peer review to educate fellows through legitimate participation while contributing to the editorial process of the journal. The latter serves as the basis of this study.

**Methods:**

The impact of the MPR program on the Special Issue’s reviewer pool served as the primary outcome of this study. This was accomplished by tracking the number of participating fellowships and their graduates who subsequently provided independent peer reviews for the sponsoring journal. Comparisons of MPRs generated by education fellows and their fellowship directors to those generated by our nonmentored faculty reviewers served two secondary outcomes: the quality of peer reviews and time from peer review offer to submission of the requisite peer reviews.

**Results:**

Over a period of five years, 82 fellows mentored by 43 fellowship directors submitted 147 MPRs. Of the 60 MPR graduates (35%), 21 were retained as independent peer reviewers. Based on a five-point editorial scoring scale (5 being excellent) mentored peer reviews received significantly higher editorial ratings (4.13 [SD 0.8]) than nonmentored faculty reviews (traditional reviewers) (3.33 [1.04]) and independent reviews by program graduates (3.61 [0.84]] (*P* < .001). We evaluated two peer-review turnaround periods. The first was the time from the initial two offers to review until two offers had been accepted. Instances where the first two offers were randomly made to MPRs were compared to offers to nonmentored faculty reviewers. The latter group took 60.2 (32.0) days compared to the MPR group, which averaged 17.2 (16.7) days (*P* < .001). The second interval measured the time from reviewer acceptance of the peer review until they submitted their completed review. There was no difference between the time in days between MPRs (10.4 [7.9]) and nonmentored faculty (9.4 [8.1]) in submitting reviews that had been accepted.

**Conclusion:**

The mentored peer-review program contributed to increasing the Special Issue’s reviewer pool with reviews of higher quality. In addition, by inviting two mentored peer-reviewers initially as compared to nonmentored faculty reviewers, the time frame for obtaining two secured peer-reviewers was significantly reduced. These findings demonstrate that a formalized approach to learning peer review by partnering with academically oriented fellowships has the potential to improve the availability of peer reviewers, the quality of peer reviews, and efficiency in the editorial review process.

## INTRODUCTION

Peer review is critical to the integrity of the scientific process by improving the quality of and curating the best possible submissions for publication. The number of journals in academic medicine has significantly increased over the years, demonstrating exponential growth in the digital age and further increasing the demand for peer reviews.^1^–^3^ This demand, along with the lack of available formal training in peer review, has resulted in an insufficient number of qualified peer reviewers available to meet the needs of many journals.^4^–^9^ The result is often substandard reviews and delays in editorial decision-making, which frustrates authors submitting their work for publication and limits the journal’s ability to publish important studies in a timely fashion.^2^,^10^–^12^

Many peer reviewers feel that they are ill-prepared for their roles.^13^–^15^ Engagement of established reviewers has been further confounded by the perceived time commitment required for activities that are largely unrecognized and unrewarded.^15^–^19^ Authors and journals have attempted to bridge this training gap by offering lectures, workshops, just-in-time resources, and published papers.^16^,^20^–^23^ Despite these efforts, journal editors continue to struggle with limited pools of qualified reviewers.^10^–^12^

To better prepare potential reviewers, there has been a call for more reliable processes to teach skills for performing peer reviews.^13^, ^24^–^27^ A number of authors have advocated for mentored peer reviews (MPR) as an effective approach to developing the knowledge and skills required to successfully fulfill this role.^2^,^28^–^30^ Preliminary reports from those who were coached or mentored through the peer review process have demonstrated mixed results. Two studies reported that participants generally enjoyed enhanced self-efficacy and a better understanding of research methodology but did not demonstrate improved peer review quality.^28^–^30^ One journal-based program recently reported outcomes based on a six-month curriculum that paired residents or fellows with experienced mentors. They reported via a preselected case-based study followed by mentorship that the quality of the postintervention mentee’s independent review of the same manuscript significantly improved over the mentee’s independent preintervention version.^32^ While some journals are willing to provide MPR opportunities to novice reviewers, formalized journal-based programs on a larger scale are uncommon. The need for additional administrative efforts, identifying qualified mentors, creating and validating a curriculum and establishing effective mentoring relationships tends to limit such efforts.^29^–^34^

Consistent with the Council of Residency Directors in Emergency Medicine’s mission to “Lead the advancement of emergency medicine education,”^35^ a partnership was formed with the *Western Journal of Emergency Medicine* to create an annual Special Issue in Educational Research and Practice (henceforth referred to as the “Special Issue”). In 2019, the editors of the Special Issue developed a curriculum for a novel, evidence-based MPR program using Kern’s six-step approach and an educational experience grounded in legitimate peripheral participation.^36^

**Population Health Research Capsule**

What do we already know about this issue?

*There is a shortage of peer reviewers available to journals, as well as a lack of effective learning opportunities for those who want to learn this skill.*

What was the research question?

*What is the impact of a journal-based mentored peer-review program (MPR) on the editorial process of that journal?*

What was the major finding of the study?

*The MPR program increased the reviewer pool by 54, provided significantly greater quality reviews [4.13(.82)/3.33(1.04)] (P < .001), and shortened turnaround time.*

How does this improve population health?

*Increasing the quality of medical education journals’ peer review process has the potential to improve medical education and, in turn, patient care.*

Evaluating the impact of the MPR program on the Special Issue’s editorial process over its first five-year period was the objective of this study. We hypothesized a positive impact on the pool of available peer reviewers, as well as improved quality and turnaround time of the MPRs.

## METHODS

This prospective, observational program evaluation employed a quasi-experimental design with nonequivalent groups to establish a clear temporal relationship between the intervention and its outcomes. The 2020–2024 annual Special Issues editorial process served as the basis for the outcomes reported. The George Washington Institutional Review Board approved this study as exempt (NCR202845). Informed consent was given, and participation was voluntary. Efforts were made to ensure that the data and privacy were properly protected and managed.

The MPR program engaged 35 emergency medicine (EM) education fellowships over the five-year course of this investigation. These fellowships focus on developing expertise in medical education, pedagogy, instructional design and/or medical education research. Emergency medicine education fellowships are of either one or two years in duration, with the latter being more likely to have a scholarly orientation.^36^ Of the participating programs, 34 of 35 (97.1%) offer a two-year fellowship, many including a master’s degree in education. The directors of these programs are highly qualified educators and researchers, whereas their fellows typically have little experience as authors or peer reviewers.^36^

Three groups made up the pool of reviewers that were compared in this study: our traditional nonmentored faculty reviewers (controls); MPRs from participating fellowships; and graduates of our MPR program who subsequently provided independent reviews. Once a manuscript passed administrative review and received approval for formal review, the opportunity to peer review was assigned on a random basis regardless of subject matter or complexity. When MPRs were selected, the corresponding fellowship director was sent an invitation to participate by the coordinating study author (JNL). If accepted, the fellow was listed as the reviewer on the journal website to blind the editors as to whether the review was an MPR or not.

### Time to Secure Two Peer Reviewers Per Manuscript

The first period tracked was the time from the initial two offers to review until two reviewers had accepted the opportunity. The Special Issue’s standard was to allow potential reviewers two weeks to respond, with a reminder at one week. If no answer was received, another reviewer was selected. To compare nonmentored faculty reviewers to MPRs, we measured only those instances in which two nonmentored faculty or two MPRs received the initial offer. This approach was adopted as the most direct measurement of the impact of this time frame on the editorial turnaround time as well as to gain a better understanding of any delays that occurred. There were insufficient numbers of MPRs for this to occur randomly prior to 2023.

### Turnaround Time for Reviews

The second interval was the time from when each accepting reviewer (controls or MPR) was provided access to the manuscript until the submission of the completed review. Completion of peer review was requested within two weeks of gaining access to the manuscript. Extensions were provided by decision editors as needed and were counted toward measurement of this metric. This period was tracked to determine whether the collaborative interaction expected from the MPR process and subsequent submission of the reviews by the fellow would prolong this phase of the editorial process. Since collaboration is not an issue for traditional reviewers or fellowship graduates providing independent reviews, the data from these two groups regarding completion of reviews were combined.

### Review Quality

The quality of each submitted review was scored by one of two editors and one of eight section editors assigned to that manuscript. The final score for each review consisted of the mean of the editor’s and the section editor’s scores. Scoring was guided by the Special Issue’s holistic editorial scoring rubric, which uses a five-point scale with anchors for each point. As an abbreviated summary, a score of 5 represents a thorough review that could stand alone as an overall evaluation of a manuscript; 4, a review that contributes substantially to the formal decision-making; 3, meets the standard of an acceptable review by providing meaningful input to decision-making but is incomplete; 2, provides some insights but does not make a substantive contribution to decision-making; and 1, fails to provide any meaningful insights.^37^ Validity evidence supporting the use of this scale in the current population has previously been published.^37^

Editors were blinded to each other’s scores by submitting to a blinded third person.^37^ Overall review quality, as determined by mean scores and the percentage of reviews scoring greater than 4.0 (“gold standard reviews”), was compared among controls, MPR and MPR graduates. For the latter group, comparisons were made to provide an understanding of this cadre’s ability to provide quality independent peer reviews following graduation from their fellowship. The process by which MPR graduates were evaluated for the potential to serve as independent reviews upon graduation has previously been described.^36^

### Data Analysis

When comparing controls, we analyzed MPR and MPR graduates scoring of review and turnaround times via *t*-test and the Levene test of equality of variances to assess whether the variances of two or more groups were equal. If a variance was found, Cohen *d* was applied as a standardized measure of the practical significance of the observed difference: 0–0.1 = no effect; 0.2–0.4 = small effect; 0.5–0.7 = intermediate effect; and greater than 0.8 = large effect. In addition, we tracked counts, frequencies, percentages and applied associated descriptive statistics related to demographics where appropriate.

### Temporal Changes in Reviewer Pool Composition

The impact of the MPR program on the number available new reviewers was evaluated first by annually tracking the number of participating fellowship programs, MPRs, and fellowship graduates providing independent peer reviews. We also tracked the percentage of the total annual reviews represented by these groups.

## RESULTS

### Reviewer Pool Size

Over this five-year period, 43 fellowship directors served as mentors in the MPR program. This is more than the number of participating programs due to turnover in the fellowship director position. A review of the Special Issues published since its inception in 2014 reveals that only two of these mentors (4.7%) served in the role of independent peer reviews for this journal prior to their involvement in the program. Over the study period, 82 fellows participated in this study through one or more mentored peer reviews. Of the 60 fellowship graduates, 21 (35.0%) have gone on to provide independent peer reviews for this journal since graduating. Table 1 provides the annual number of MPRs and MPR graduates providing independent peer reviews, as well as their impact on the overall number of reviews and recognition as gold standard reviewers.

### Review Quality

Editorial evaluation of review quality, based on the Special Issue’s holistic editorial scoring rubric of traditional, MPRs, and independent reviews of graduates, is reported in Table 2.

The Student—Newman—Keuls (post hoc test indicated that the MPR group reviews scored significantly higher in quality than did the other two groups (nonmentored faculty and MPR graduates). Furthermore, there was no difference between the quality scores of nonmentored faculty reviewers and those submitted by graduates of the MPR program. (Table 2)

### Time to Secure Two Peer Reviewers

The first editorial period assessed was from the time when the first offers were made until two review opportunities were accepted in instances when the first two were MPRs versus two control reviewers were compared (Table 3).

The difference between the means of the two groups was 43 days *(P < .*001). Issues that led to delays in obtaining two acceptances are highlighted in Figure. Note the number of “no answers” and “refusals” in comparing the two groups and the delays.

### Turnaround Time for Reviews

After accepting the review, the next period tracked was from the time in days—from gaining access to the manuscript until the review was submitted to the journal. The mean turnaround time in days for controls (*n* = 305) was 9.4 (8.1) days versus 10.4 (7.9) days for MPRs (*n* = 138). There was no statistical difference between the two groups (*P* = .21).

## DISCUSSION

The MPR program was initiated five years ago as a professional development opportunity for education fellows. Since that time, the program has had a significant effect on the Special Issue’s peer-review process by the increasing number of participating programs and their graduates who subsequently provide independent reviews. This growth continues to increase annually. Secondly, the participating MPR programs provided higher quality reviews than did our traditional nonmentored faculty. Finally, the MPRs had a shorter turnaround time of peer reviews, which facilitated timely editorial decision-making.

The impact of the MPR program on the growing number of peer reviewers is the result of several factors. First, the number of participating education fellowship programs grew from 14 to 35 programs over the five-year period since the program’s inception. A total of 41 fellowship directors recognized the value of this skill-development opportunity for their fellows and thus chose to participate in the Special Issue MPR program. This participation has resulted in a reliable cadre of high-quality reviewers for the Special Issue. Over the past three years, the MPRs have been responsible for nearly 50% of the peer reviews required by the Special Issue. Over this same period, the number of manuscript submissions decreased, limiting our ability to expand opportunities to interested fellowships or to fully realize the potential value of this initiative to participants and the journal. A second way in which the MPR program has contributed to the pool of peer reviewers is by offering graduates the opportunity to provide independent peer reviews on which they continue to receive feedback based on the decision editor’s summary and the second reviewer’s comments. The number of such reviewers has grown substantially and currently represents 35% of graduates. Based on this growth, MPR graduates who provide independent reviews appear to have the greatest potential to contribute to the long-term expansion of the reviewer pool.

The MPR program was developed as an experience for early-career scholars to promote skills related to peer review. Such experiences have the potential to be adopted by fellows and integrated into their own practices, further improving their knowledge and skills related to education scholarship.^38^ Over the past two years, six prior fellow mentees have stepped forward to serve as mentors in the MPR program, primarily as fellowship faculty. These observations suggest that this may well serve as a scaling factor that further contributes to the pool of trained peer reviewers over time.

Based on average review scores and the percentage of those who are recognized as gold standard reviewers, the quality of mentored peer reviews was significantly greater than that of our traditional reviewer pool (nonmentored faculty). This finding runs contrary to the findings of a previous MPR study in which the sponsoring journal paired new reviewers with high-functioning senior reviewers.^33^ The mentorship suggested by the sponsoring journal was to discuss the review with the mentees by “email or phone” followed by the mentor providing feedback to the mentee. After reviewing the literature, these authors hypothesized that the lack of improvement from this experience was likely due to an insufficiently focused and detailed mentoring process between individuals with no prior or ongoing relationships. Due to their prior training and experience in education and scholarship, education fellowship directors are likely a different group than our traditional faculty reviewers. Considering the novice status of most participating fellows in the MPR program,^36^ the quality of MPRs likely resulted from a substantive mentorship in the context of a longitudinal mentoring relationship integrated into fellowship training. An additional contributing factor may also include the collaborative approach of MPRs.

The quality of independent peer reviews by fellowship graduates was found to be equivalent to that of traditional reviewers. Considering participating fellows’ relative lack of experience upon beginning fellowship,^36^ this performance appears to reflect substantive growth in the fellows’ skills and knowledge related to education scholarship and peer review based on their fellowship experiences. For most, it is doubtful that two years of fellowship would prepare fresh graduates, one to two years out of training, to provide the same quality of peer review as those who were mentored by their fellowship director. This decline in quality based on editor scoring appears to reflect the loss of mentorship and perhaps also the opportunity to collaborate.

Authors consider multiple factors when choosing a journal for manuscript submission. These include journal reputation, impact factor, relevance of the topic, target audience, and timeliness of turnaround time from submission to final decision and publication.^39^,^40^ Although all are important, the latter is the one over which editors have the most direct control. Consequently, our finding that inviting two MPRs initially, as opposed to two traditional reviewers, reduced the time to obtain two accepted reviews by six weeks is potentially important. As demonstrated in Figure, the delays in recruiting traditional nonmentored peer reviewers versus MPRs was largely based on the number of “no responses” and “refusals.”

Consistent with our findings, a recently published preliminary report by a journal-based MPR program demonstrated a reduction in ignored and declined peer-review requests, thereby reducing turnaround times for reviews when comparing MPRs to traditional reviewers.^12^ This disparity is likely the result of the different values these two groups place on this experience. Nonmentored faculty reviewers have competing priorities that are often perceived as having a greater impact on their career, making it less likely that they accept an offer to review a manuscript.^13^–^15^,^17^–^19^ In contrast, we reported previously that fellows value MPRs as professional development opportunities and that fellowship directors see this as an important addition to the training they provide.^36^

Fellows generally have more time for a jointly performed exercise that supports their education, which may also be a contributing factor. However, once the opportunity to review was accepted, there was no difference between the two groups in the time to submit the completed review, thereby demonstrating a commitment to the task at hand by both groups once they had committed to the review.

## LIMITATIONS

The Special Issue accepts submission types in all standard educational categories (original research, educational advances, reviews, and scholarly perspectives). As submissions were randomly assigned to peer reviewers, there is always some risk of unequal distribution of manuscript types. Although this limitation should be kept in mind, it appears that the higher quality reviews of MPRs scale to more complex studies and manuscript types.

Although blinding of the editor’s score occurred by having the fellow listed on the website, there was potential for the editors to identify the fellows and nonmentored faculty reviewers. The medical education community in EM is such that it is not uncommon to recognize the names of other faculty members and learners from prior teaching/mentoring experiences. This raises the potential for bias based on the recognition of both nonmentored faculty reviewers and fellows involved in mentored peer reviews. It is also possible that the editor who participated in inviting fellowship directors, traditional reviewers, and graduates to review could have recognized submitting reviewers. Blinding of the review scores by the section editor and the guest editor was intended to mitigate such bias.

The success and thus generalizability of this journal-based program is largely dependent on integrating this experience into the curriculum of a specialty fellowship related to scholarship. This educational environment set the stage for quality mentorship and significant motivation of mentors/mentees. The absence of educational support, in this case provided by fellowships, is a major obstacle to a successful journal-based mentored peer review program.^32^,^33^ Although not measured in this study, there is no question that establishing and maintaining this MPR program for the Special Issue with a limited number of annual submissions (65–160) requires a substantive investment of time and administrative support. Such resources may not be available to journals with limited staff or resources.^33^

Other factors that may affect success include the culture and mission of the journal and the degree to which they have access to a “fellowship” pool. Finally, the generalizability of our findings to journals with a much larger annual submission rate may be restricted by the limited impact of such a program on their editorial process.

## CONCLUSION

The development of a formal educational opportunity in mentored peer reviews for emergency medicine education fellowships has had a significant impact on our editorial review process. The size of our reviewer pool continues to increase annually in a manner that appears sustainable. Peer-review quality improved, while editorial review turnaround times decreased.

## Figures and Tables

**Figure:**
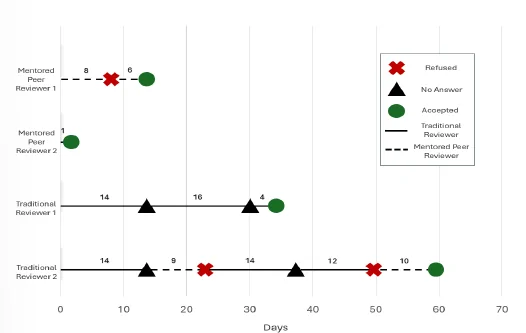
Comparison of the times (days) and steps in obtaining two acceptances when the first two offers were nonmentored faculty versus two mentored peer-reviews (MPRs). The examples used were that instance in each group that came closest to that group’s mean time (nonmentored faculty, 57; and MPRs,15 days).

**Table 1:** Indices regarding the impact of a mentored peer-review program on the editorial review process of the Special Issue in Educational Research and Practice over the five-year study period.

Issue year	2020	2021	2022	2023	2024
Education Fellowships	14	19	27	30	35
MPRs (% Total Reviews)	9.7% (16/165)	29.5% (38/129)	42.1% (32/83)	47.4% (27/57)	49.3% (34/69)
MPRs (% of All Gold Standard Reviews)	50.0% (10/20)	35.3% (6/17)	81.0% (17/21)	82.4% (14/17)	54.5% (12/22)
Graduates (Reviewers)	0	1	2	12	21
Graduates (% Total Reviews)	0.0% (0/165)	0.7% (1/129)	2.4% (2/83)	21.1% (12/57)	30.4% (21/69)
Graduates (% of Gold Standards Reviewers)	0.0% (0/20)	5.0% (1/17)	4.8% (1/21)	11.8% (2/17)	18.2% (4/22)

Graduates represent prior MPR participants providing independent peer review following graduation.

Gold standard review status is achieved by a blinded editor’s scoring of > 4.0 on a 1–5 scale ( 5 being excellent).

*MPRs*, mentored peer reviews by education fellows.

**Table 2:** Comparison of editorial scoring of reviews (5 being exceptional) among controls’ peer reviews, mentored peer reviewers (MPRs), and graduates of the mentored-peer review program (based on their average editorial review rating.

Groups (*n*)	Mean Review Score	Std Deviation	*S-N-K Post Hoc	*F* ratio	*P* value
Traditional (292)	3.33	1.04		61.243	< .001**
MPR Graduates (30)	3.61	0.84			
MPRs (141)	4.13	0.82	a		
Total (463)	463	1.03			

There is a significant difference at the *P* < .001*, and the Levine test of equality of variances (effect size) was significant when comparing mentored peer reviews from the other two groups.

* Cohen *d* measurement of variance effect size was intermediate to large (.7–.8). The Student-Newman-Keuls post hoc test indicated that the traditional and program graduates were statistically similar while MPRs were statistically different^a^ than both other groups at the *P* < .05 level.

*MPRs*, mentored peer reviews of fellows; *MPR Graduates*, independent reviews by previously participating fellowship graduates; *S-N-K*, Student-Newman-Keuls.

**Table 3:** Comparison between instances in which two traditional reviewers or two mentored peer reviewers (2023 and 2024) were the first two offers to review and tracking the turn-around time from initial peer-review requests to that point in time when two offers were accepted.

Reviewer Type	Number of Manuscripts	Mean Turn-around Time Days (+/−SD)	t*	*P* value**	ES
Nonmentored faculty	17	60.2 (32.0)	4.95	< .001	1.70
MPRs	18	17.2 (16.7)			
TOTAL	35	38.1 (33.1)			

* Because the Levene test for equality of variances was significant, equal variances between groups was not assumed; therefore, the smaller (adjusted) *t*-value and associated degrees of freedom were selected. The *t*-value adjusted for unequal variances was significant at the **P < .001 level. The Cohen *d* effect size for review ratings between MPRs and controls reviewers is considered very large.

*ES*, effect size; *MPRs*, mentored peer reviews; *SD*, standard deviation.
